# A Case of Orbital Myiasis in Recurrent Eyelid Basal Cell Carcinoma Invasive into the Orbit

**DOI:** 10.1155/2016/2904346

**Published:** 2016-08-09

**Authors:** Triptesh Raj Pandey, Gulshan Bahadur Shrestha, Ranju Kharel (Sitaula), Dev Narayan Shah

**Affiliations:** Institute of Medicine, B.P. Koirala Lions Centre for Ophthalmic Studies, Maharajgunj, Kathmandu, Nepal

## Abstract

*Introduction*. Orbital myiasis is the infestation of the orbital tissues by fly larvae or maggots. Compromise of periorbital tissues by malignant disease, surgery, ischemia, or infection may predispose the patient to orbital myiasis.* Case Report*. A 73-year-old male patient with neglected recurrent basal cell carcinoma of the eyelid invasive into the orbit presented with complaints of intense itching and crawling sensation with maggots wriggling and falling from the wound of left orbit. The patient improved following manual removal of the maggots along with oral Ivermectin treatment. Recurrence of the basal cell carcinoma was confirmed by punch biopsy from the wound and extended exenteration of the orbit followed by reconstructive surgery was done.* Conclusion*. Orbital myiasis is a rare and preventable ocular morbidity that can complicate the malignancies resulting in widespread tissue destruction. The broad spectrum antiparasitic agent, Ivermectin, can be used as noninvasive means to treat orbital myiasis. In massive orbital myiasis and those associated with malignancies, exenteration of the orbit must be seriously considered.

## 1. Introduction

Myiasis is defined as the infestation of living tissue of human and other vertebrate animals by fly larvae or maggots [1]. Large numbers of larva invading and destroying orbital contents cause orbital myiasis in human. However, orbital myiasis is a very rare entity with very few reports having been published worldwide. It accounts for less than 5% of the cases of human myiasis [2]. Crowded conditions, debility, low socioeconomic status, and poor personal hygiene are the predisposing factors [3]. Compromise of periorbital tissues by malignant disease, surgery, ischemia, or infection may predispose the patient to orbital myiasis. Management of orbital myiasis may range from simple manual removal of the maggots to destructive surgeries of the globe and orbit [4]. In recent years, Ivermectin, has been successfully used as a noninvasive means to treat orbital myiasis [5]. The authors herein report an extremely rare case of orbital myiasis in a recurrent basal cell carcinoma of eyelid invasive into the orbit.

## 2. Case Report

A 73-year-old male from a remote village presented with complaints of intense itching and crawling sensation with maggots wriggling and falling from the wound of left orbit since 1 week. This was associated with pain and foul-smelling purulent discharge from the wound. He had a history of surgical removal of the pigmented nonhealing ulcerated lesion in left lower eyelid 9 years ago, reported as basal cell carcinoma.

At presentation, the unaided visual acuity was 6/18 and best corrected visual acuity was 6/9 in right eye. The rest of the findings in the right eye was normal. In left orbit examination, there was a large fungating, foul-smelling, ulcerated mass with wound extension up to eyebrow superiorly and malar region inferiorly and up to lateral canthus and medial canthus. Necrotic tissues along with crusts and scabs were present within and at the wound edges along with granulation tissues. There was intense foul smell and purulent discharge with multiple maggots crawling over the wound. The left eyeball was not seen.

**Figure 1:**
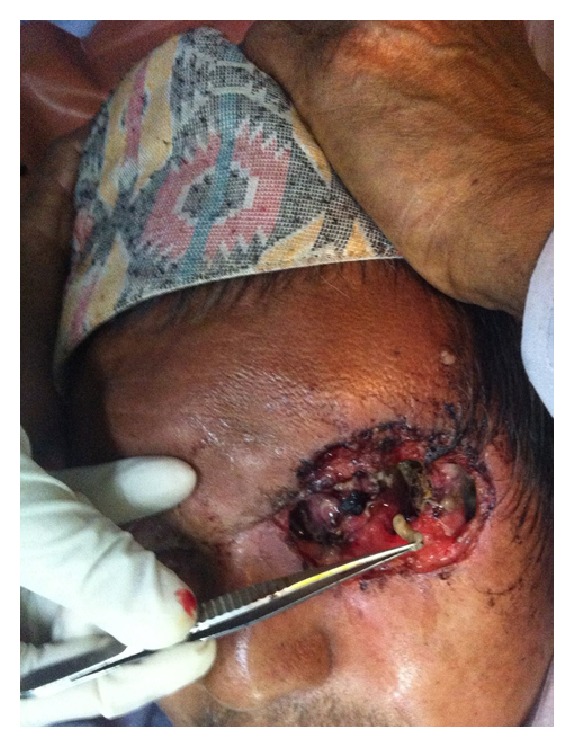
Clinical photograph showing extent of the wound and manual removal of a maggot.

Initially, around 100 maggots were manually removed and debridement of necrotic tissues was done under local anesthesia (Figure 1). Exposed bone edges of lateral wall of the orbit were noticed after sterile dressing. The patient was started on oral antibiotics and analgesics. He also received single dose of oral Ivermectin (9 mg, 200 mcg/kg) following which the patient had significant relief from pain and discomfort. Dead maggots were easily removed from the wound and the rapid wound healing ensued. The left socket was covered with healthy granulation tissues and was maggot-free within few days.

**Figure 2:**
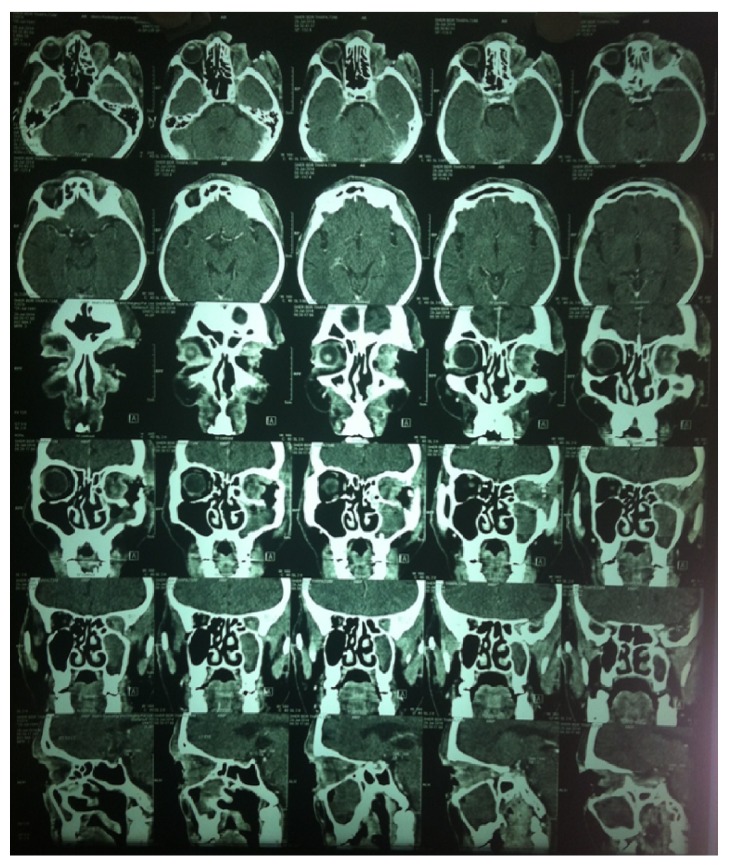
Axial and Coronal section CT scan showing soft tissue density area in left maxillary sinus and left orbit with erosion of the floor and lateral wall of the orbit. No intracranial abnormality seen.

The CT scan of orbit and paranasal sinuses revealed left anophthalmic socket with soft tissue lesion in left orbit and maxillary sinus along with erosion of orbital floor and lateral wall of the orbit (Figure 2). However, orbital apex and cranial fossa were not involved. Nasal endoscopy revealed only streaks of mucopurulent discharge in maxillary sinus. Punch biopsy was done from the wound margins and central region and sent for histopathological evaluation. The biopsy report revealed basal cell carcinoma invasive into the left orbit.

**Figure 3:**
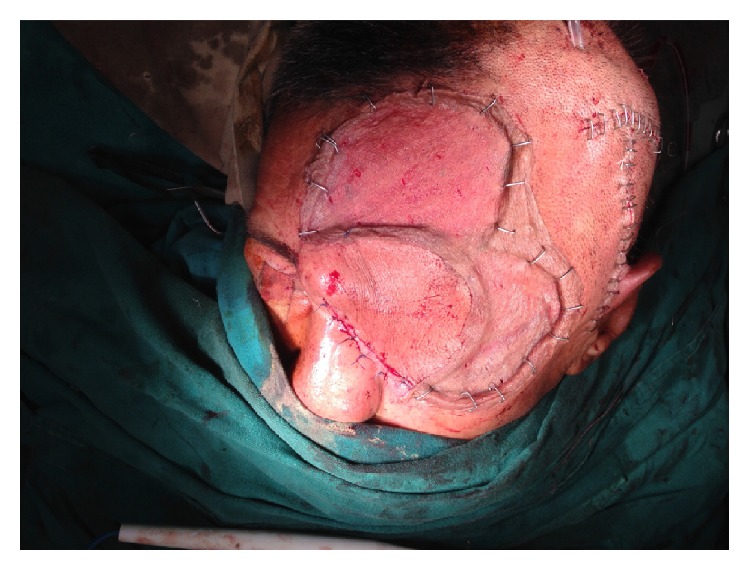
Clinical photograph showing postoperative status following extended exenteration and myocutaneous flap transposition from forehead and temporal region to cover the wound defect and left orbit.

Hence, the patient underwent uneventful extended exenteration of the left orbit and the socket was covered by transposition of myocutaneous flap from forehead and temporal region (Figure 3). The postoperative period was also uneventful and patient recovered well. The patient was reassessed 7, 14, 30, and 60 days after the discharge from the hospital with complete healing of the wound.

## 3. Discussion

The term “myiasis” is derived from the Greek word “Myia” meaning fly. Myiasis is defined as the infestation of living tissue of human and other vertebrate animals by fly larvae or maggots [1]. Ophthalmomyiasis refers to infestations of the eye and/or ocular adnexa. In humans, the orbital form of ophthalmomyiasis is particularly serious [2]. Large numbers of larva invading and destroying orbital contents cause orbital myiasis in human. Keyt first reported it in 1900 (AD) and later on, Elliot reported it from India in 1910 (AD) [6]. Orbital myiasis is a very rare entity with very few reports having been published worldwide. It accounts for less than 5% of the cases of human myiasis [2]. It can occur in most regions of the world, particularly in rural areas of underdeveloped countries where the standard of hygiene is low and flies abound [6]. Compromise of periorbital tissues by malignant disease, surgery, ischaemia, or infection may predispose the patient to myiasis. Crowded conditions, debility, low socioeconomic status, and poor personal hygiene are the predisposing factors [3]. The orbital myiasis occurs commonly in neglected orbital wound, which can cause massive destruction of orbital tissues, accompanied by severe inflammations and secondary infections [2]. In extreme cases, the larvae can cause destruction beyond the orbit with mucosal sinuses and intracranial invasion which can be life threatening. Cases of neonatal fatal cerebral myiasis, caused by the penetration of larva through the fibrous portion of the fontanel, have been reported [7].

In the presented case report, the patient developed a secondary orbital myiasis in neglected wound of recurrent basal cell carcinoma of the eyelid invasive into the orbit. The main predisposing factor for the maggot infestation in our patient was probably the large bed of necrotic tissue offered by the basal cell carcinoma. Poor general health status, lack of self-care and communication, lack of immediate medical care in rural area, and an overcrowded living environment were other possible factors. There are other case reports of the orbital myiasis complicating basal cell carcinoma [8, 9].

The contrast enhanced computed tomography or magnetic resonance imaging of orbit and brain is useful for delineating the extent of orbital involvement and excluding the intranasal and intracranial spread [10]. Management of orbital myiasis may range from simple manual removal of the maggots to destructive surgeries of the globe and orbit [4]. The factors that affect the outcome and success of treatment of orbital myiasis depend upon extent of invasion and underlying predisposing conditions. The key step in the management of the less extensive orbital myiasis should be directed toward the manual removal of all the invading organisms. Solutions hydrogen peroxide, chloroform, ether, ethanol, and turpentine have been used to facilitate the easy removal of the maggots [2]. Controlling the secondary infection in orbital myiasis is of utmost importance. In cases of extensive orbital myiasis and those associated with malignancy may require exenteration to prevent intracranial extension of the tissue destruction [1]. In recent years, broad spectrum antiparasitic agent Ivermectin has been successfully used as a noninvasive means to treat orbital myiasis. Use of Ivermectin therapy has been recommended prior to surgical debridement to prevent destructive surgery and to reduce the difficulty associated with mechanical removal of the maggots with massive orbital invasion [5]. In the reported case, the patient had significant relief from pain and discomfort following oral Ivermectin administration. It also facilitated the easy removal of the maggots from the wound and promoted rapid wound healing. In case of extensive orbital myiasis and those associated with malignancy, exenteration may be required to prevent intracranial extension of the tissue destruction [1]. Likewise, in our case, the patient had recurrent basal cell carcinoma invasive into the orbit, so the patient underwent extended exenteration of the left orbit followed by transposition of the myocutaneous flap from forehead and temporal region to cover wound defect of the left orbit.

## 4. Conclusion

Orbital myiasis is a rare and preventable ocular morbidity that can complicate the orbital malignancies resulting in widespread tissue destruction. Controlling fly population, educating patients with advanced malignancy about maintaining hygiene can prevent orbital myiasis. The broad spectrum antiparasitic agent, Ivermectin, can be used as noninvasive means to treat orbital myiasis. In massive orbital myiasis and those associated with malignancies, there is the possibility of intranasal and/or intracranial extension due to the close proximity of the vital structures, rendering this as a potential life threatening condition. In those cases, exenteration must be seriously considered.
